# An Assessment of Physical Activity and Risk Factors in People Living with Dementia: Findings from a Cross-Sectional Study in a Long-Term Care Facility in Vietnam

**DOI:** 10.3390/geriatrics9030057

**Published:** 2024-04-29

**Authors:** Khanh Nam Do, Linh Thao Thi Le, Son Cong Dang, Ha Thu Thi Nguyen, Giang Thu Nguyen, Hang Van Thi Ngo, Huong Lan Thi Nguyen, Lieu Thu Thi Nguyen, Anh Kim Dang, Huong Thi Le

**Affiliations:** 1 School of Preventive Medicine and Public Health, Hanoi Medical University, Hanoi 100000, Vietnam; donamkhanh@hmu.edu.vn (K.N.D.); son.ipmph@gmail.com (S.C.D.); nguyenthithuha@ipmph.edu.vn (H.T.T.N.); huonglandd@hmu.edu.vn (H.L.T.N.); nguyenthulieu@hmu.edu.vn (L.T.T.N.); dangkimanh@hmu.edu.vn (A.K.D.); lethihuong@hmu.edu.vn (H.T.L.); 2 Population Health Sciences Institute, Faculty of Medical Science, Newcastle University, Tyne NE2 4HH, UK; giang.nguyen@newcastle.ac.uk; 3 VinUniversity Medical Simulation Center, College of Health Sciences, VinUniversity, Hanoi 100000, Vietnam; vanhangngo@gmail.com; 4 Department of Nutrition, Saint Paul General Hospital, Hanoi 100000, Vietnam; 5 Department of Nutrition, National Hospital of Obstetrics and Gynecology, Hanoi 100000, Vietnam; 6 Queensland Alliance for Environmental Health Sciences (QAEHS), The University of Queensland, 20 Cornwall Street, Woolloongabba, Brisbane 4102, Australia; 7 Department of Nutrition and Dietetics, Hanoi Medical University Hospital, Hanoi 100000, Vietnam

**Keywords:** physical activity, exercise, care dependency, dementia, malnutrition, activities of daily living, MMSE, CDS, IPAQ, MET

## Abstract

Background: People living with dementia (PLWD) may experience substantial cognitive decline as the disease progresses, which interferes with their daily activities. This study aimed to assess physical activity (PA) performance and care dependency (CD) and identify factors related to PA among PLWD. Methods: We conducted a cross-sectional study in 63 PLWD from National Geriatrics Hospital, Vietnam, from 2021 to 2023. We used the Mini Nutritional Assessment (MNA), International Physical Activity Questionnaire (IPAQ), and Care Dependency Scale (CDS) to assess the nutritional status and the levels of PA and CD, respectively. We used the Mann–Whitney test to compare the differences in the PA types and CD levels between dementia levels and a multivariable logistics regression model to analyze factors related to PA. Results: More than half of the subjects had mild dementia. In total, 35% of the PLWD had a low level of PA, and 46.3% were completely independent of care. The mean score in each CDS aspect of the subjects with moderate/severe dementia was statistically significantly lower than that of those with mild dementia (*p* ≤ 0.05). Lower dependency (OR = 0.9; 95% CI = 0.88–0.99) and malnutrition (OR = 15.4; 95% CI = 1.18–20.21) were associated with insufficient PA in the PLWD. Conclusion: Formal caregivers and healthcare workers should encourage PLWD to perform physical activities at any level and personalize the development of tailored and nutritional care strategies for each individual.

## 1. Introduction

Dementia is a gradual decline in cognitive ability across various areas, such as memory, learning, orientation, language, comprehension, and judgment. These declines are significant enough to disrupt daily functioning [1,2]. According to the World Alzheimer Report 2015, it was estimated that 36 million people were living with dementia in 2010; this number is projected to increase to 66 million by 2030 and 115 million by 2050, with the rate of increase nearly doubling every 20 years [3]. Dementia is caused by multiple factors. According to an independent report by the US National Institutes of Health, seven characteristics have consistently been shown to contribute to cognitive decline [2]. These factors are diabetes mellitus, smoking, depression, lack of cognitive stimulation, lack of physical activity (PA), and a poor diet characterized by high saturated fat and low vegetable intake [2,4].

An active lifestyle is associated with brain health. The most intense levels of physical exercise offer the greatest protection [5,6]. Engaging in PA positively impacts the brain’s structures, possibly explaining the observed connections [7]. Physical inactivity is recognized as one of the primary contributors to dementia, constituting around 13% (almost 4.3 million) of the global incidents of dementia [4]. Several epidemiological studies have established a strong correlation between cognitive impairment and engagement in PA [8,9]. Rosano et al. (2005) indicated reduced levels of PA, including walking, concurrently with a deterioration in cognitive function [8]. Mobility impairments and diminished performance in activities of daily living (ADL) are prevalent among those with dementia [10,11,12]. Due to cognitive and functional decline, people living with dementia (PLWD) have demonstrated certain deficits in motor skills, including reduced levels of flexibility, agility, strength, balance, and aerobic resistance [13,14,15]. As a result, physical inactivity and dementia form a reciprocal relationship in which they impact each other.

An important risk factor for encountering falls is cognitive impairment [16]. Falls are a common symptom of dementia, especially in the elderly, but they can also develop in younger people. Indeed, there is an increased risk of falls in adults with cognitive impairment compared to the cognitively healthy population as a whole [17]. Falls occur annually for 60–80% of PLWD [18]. According to a systematic review and thematic synthesis of the qualitative literature by Franco et al., (2015) [19], 28% of the studies showed that older individuals were concerned about falling and sustaining major injuries during PA, leading to a lack of confidence in exercising independently. In addition, previous falls made PLWD more anxious about exercising [19]. Home care for older adults with dementia, as well as the prevention of falls within this population, are both greatly impacted by the involvement of carers [20]. As a result, older people become more dependent on their caregivers and minimize intense physical exercise, which can have serious effects on those with dementia; meanwhile, engaging in PA and doing exercise at any level can improve cognitive performance in PLWD [21].

Vietnam is undergoing a significant demographic change [22], characterized by a significant increase in the percentage of over-60s in recent decades [23]. One study in Vietnam found that about 10% of the older participants expressed a requirement for assistance with ADL. Additionally, more than two-thirds of the older individuals in rural Vietnam indicated a need for support with instrumental or intellectual ADL [24]. The older population, especially PLWD, have difficulties with ADL including domestic chores, personal hygiene, food, dressing, eating, and bathing [25]; thus, receiving support from caregivers is particularly important for them to maintain a normal life.

Current studies in Vietnam are primarily concerned with evaluating the mental health of older individuals [26,27], along with the quality of life and the demand for healthcare [24,28,29,30,31]. To comprehensively assess the health status of older people, especially those with dementia, it is crucial to gain an understanding of the physical health and nutritional status as well as the care dependency among them. In addition, the understanding of these aspects in Vietnam is limited [30,32,33]. Therefore, this study aimed to assess the physical activity performance and care dependency among PLWD and identify related factors.

## 2. Subjects and Methods

### 2.1. Study Subjects

We conducted a cross-sectional study at the National Geriatrics Hospital (NGH) in Hanoi, Vietnam, from April 2021 to April 2023. Participants were recruited from NGH’s outpatient and inpatient clinics. We selected patients who (1) were diagnosed with dementia according to the *Diagnostic and Statistical Manual of Mental Disorders, Fifth Edition* (DSM-V) criteria (2013) [34] by doctors/specialists; (2) were receiving a treatment regimen and had full medical records accessible at the study setting; and (3) were made fully aware of the study and opted to participate voluntarily or their families or formal caregivers provided consent. The exclusion criteria specified people who (1) had at least one disease that could negatively impact their nutritional status, such as fever, cancer, acute illness, severe food allergy, or severe digestive disorder; (2) had a major mental illness or neurological disorder other than dementia that could have negatively impacted their sense of smell and taste or had developmental abnormalities; or (3) were on a special diet.

### 2.2. Sample Size and Sampling Technique

We applied the sample size formula to estimate a proportion. Based on a statistical significance level (α) of 0.05, a hypothesized proportion (p) of 0.76 (the proportion of PLWD who suffered from malnutrition and were at risk of malnutrition according to MNA score in a study in Belgian hospitals [35]), a relative deviation (ε) of 0.15, and an additional 20% of the sample for decline response, a minimal sample size of 63 subjects was indicated. Patients who attended the hospital during the study period and matched the above criteria were chosen until the sample size of 63 was reached.

### 2.3. Measurements and Instruments

Interviewer-administered questionnaires were designed using REDCap 14.3.1 software to collect the study data. The questionnaire was divided into four main sections:

(1) ***Socio-demographic characteristics*** included information on age, gender, living area, educational attainment, and occupation.
(2) ***Clinical data*** included dementia severity levels assessed using the Mini Mental State Exam (MMSE). The MMSE had a total of 30 questions with a range score of 0 to 30, and each correct answer was given 1 point, with 0 to 9 points indicating severe cognitive impairment, 10 to 17 indicating moderate impairment, 18 to 23 indicating mild impairment, and ≥24 indicating normal cognitive abilities [36]. We categorized the patients who participated in this study into two groups: mild dementia (MMSE score ≥ 18) and moderate/severe dementia (MMSE score ≤ 17). Nutritional status was assessed using anthropometric measurements and the Mini Nutritional Assessment—Long Form (MNA-LF). Anthropometric indicators covered weight, height, body mass index (BMI), and body fat percentage (BF%). Weight and BF% were estimated using the Tanita scale’s bioelectrical impedance analysis (BIA) (BC 758). The vertical height of patients was measured using a wooden ruler with an accuracy of 1 mm. The metric system calculated BMI by dividing weight (kg) by squared height (m). The MNA-LF considered dietary intake, reduced weight, and physical and psychological issues over the last three months, with two sections as screening and assessment. The total score ranged from 0 to 30, with <17 points indicating malnutrition, 17–<24 indicating at risk of malnutrition, and 24–30 indicating normal nutritional levels [37].
(3) ***Physical activity (PA) levels*** of PWD were determined using the International Physical Activity Questionnaire—Short form (IPAQ-SF). The IPAQ-SF consists of 7 questions related to the number of days of the week and the average number of hours–minutes in the day performing vigorous, moderate activities and walking (time for activities is calculated when performed at least every 10 min) [38]. The estimated energy expenditure (MET) estimates the kilocalories per kilogram used per day (kcal × kg^−1^ × d^−1^). The physical exercise dose is estimated in METs per minute per week (METs/min/week) [39]. The metabolic equivalent task (METS/min/week) of each PA domain was calculated as duration (weekly minutes spent) × frequency per week × MET intensity (8 METs for vigorous activities, 4 for moderate activities, and 3.3 for walking). Energy expenditure for each individual was obtained by summing these three activity domains. The scoring protocol was based on Guidelines for Data Processing and Analysis of IPAQ [40].
(4) ***Care Dependency Scale (CDS)*** covers 15 aspects; each aspect has a brief description and five care-dependent criteria, including 1: completely care-dependent; 2: care-dependent to a great extent; 3: partially care-dependent; 4: care-dependent to a limited extent; and 5: almost care-independent. After calculating the total score, each patient would then be assigned to one of three levels of dependence, in which the lower the point, the more dependence (15–44: high care dependency, 45–59: medium dependency and 60–75: low dependency) [41].

### 2.4. Data Analysis

After collecting data via REDCap Mobile App v5.27.2 software, they were analyzed using Stata 16.0 software. We describe quantitative variables using the mean and standard deviation (SD) and qualitative variables using frequency and percentage (%). We used the Mann–Whitney test and *t*-test to compare the differences in physical activity types and energy expenditure (MET) between two groups of MMSE. We also applied a multivariable logistics regression model to analyze factors related to the low level of PA. A *p*-value ≤ 0.05 was considered statistically significant for all analyses.

### 2.5. Ethical Considerations and Research Funding

At the beginning of data collection, written informed consent was provided by patients or their families/formal caregivers. Participants could withdraw from the study at any time, and their participation did not impact on patients’ treatment progression. All information on research subjects was for research purposes only and guaranteed to be kept confidential. This study was supported by the National Institute on Aging (NIA) of the National Institutes of Medicine (NIH) at R01AG064688 (Hinton/Nguyen MPI). The content of this study is solely the author’s responsibility and does not necessarily represent the official views of the NIA and NIH.

## 3. Results

Table 1 shows the general characteristics of the study subjects. Among the 63 PLWD, the majority were female (68.2%), 98%were retired, 93.6% were currently living with family, and 74.6% were living in urban areas. Those with less than a high-school-level education (41.3%) made up the largest proportion of educational attainment. The mean age of the study population was 74.7 ± 7.3 (years), and the total MMSE score was 17.1 ± 7.0. According to the assessment of the dementia severity levels, 52.4% of the participants had mild dementia and 47.6% had moderate or severe dementia. Regarding the nutritional status, 74.6% exhibited malnutrition or were at risk of malnutrition.

The physical activity levels expressed by the dementia severity levels are shown in Table 2. Among the 63 PLWD, 35% were defined as having a low level of physical exercise, and none achieved a high level of physical activity. In total, the mean of minutes per week of walking, moderate-intensity activity, and vigorous-intensity activity were 856.3 (SD = 985.0), 414.1 (SD = 296.0), and 135.0 (SD = 21.2), respectively (Table S1). However, the differences in the two dementia groups’ physical activity time per week were not statistically significant. Regarding the energy expenditure (MET), all the participants’ MET-minutes/week of total physical activity was 2476.4 (SD = 3412.5).

**Figure 1:**
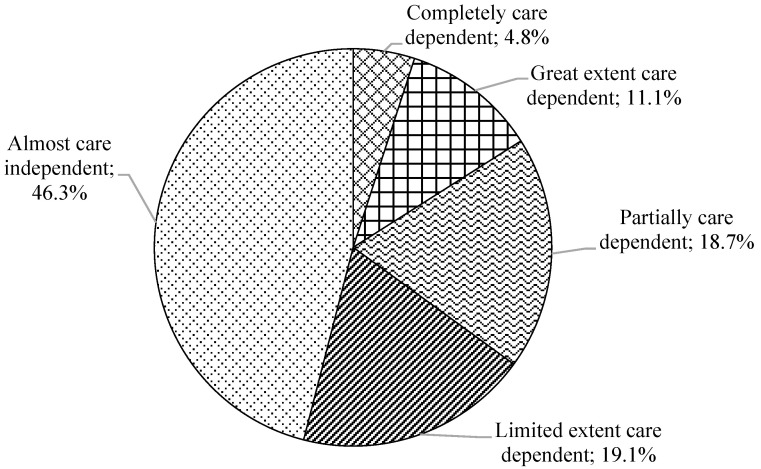
The percentage of care dependency levels of patients with dementia (n = 63).

Figure 1 illustrates the distribution of the care dependency levels across all the participants according to the Care Dependency Scale (CDS). In total, 4.8% and 11.1% of the PLWD reported being completely care-dependent and care-dependent to a great extent, respectively. The percentage of the participants who were partially and, to a great extent, care-dependent was similar (approximately 19%). Nearly half of the participants (46.3%) were completely care-independent. The total CDS score was 61.3 (SD = 16.7), in which the lower the score, the more dependent on care a patient was. The mean score of 15 components ranged from 3 to 5. The average score of each component of the CDS in the participants with moderate and severe dementia was significantly lower than those with mild dementia (Table S2).

Table 3 presents the factors related to low physical activity levels among the PLWD in Vietnam. The participants with higher CDS scores or lower dependency were less likely to exhibit low physical activity (OR = 0.9; 95% CI = 0.88–0.99). In addition, being malnourished or at risk of malnutrition was positively associated with exhibiting insufficient physical activity (OR = 15.4; 95% CI = 1.18–20.21).

## 4. Discussion

Our study is one of the first to evaluate the levels of PA and related factors, including the CD and nutritional status, of PLWD in Vietnam. The findings from this study are essential for medical professionals and caregivers to understand the PA levels of PLWD and to develop appropriate intervention strategies. We found that a high percentage of the participants were classified as being physically inactive. Higher levels of CD and being malnourished/at risk of malnutrition were factors that positively related to the low PA level among the PLWD.

In this study, a high percentage of the participants exhibited a low PA intensity. This result can be attributed to the deterioration of functional and health status associated with aging [24]. This process is linked to physiological changes that lead to decreased functional ability, body composition modifications, increased risk for chronic diseases, and reductions in PA amount and intensity in the older population [42]. Our study participants were older hospital-based patients experiencing cognitive issues, which may prevent them from engaging in vigorous activity. Our results concur with those of a study by Elena de Dios-Rodríguez et al. (2023) [43], which depicted that the highest percentage of the PLWD engaged in moderate PA. Despite the prevalence of light activities, the average energy expenditure of our study subjects (METs/min/week) was approximately two times higher than that of subjects from prior studies conducted in Spain and the US (with average METs of 1052.47 and 1071.3, respectively) [43,44]. According to Telenius (year), for many people, going for a daily stroll was a significant ritual that added significance to their day [45]. Rafferty et al. (2002) indicated that the PAs that older people tend to prefer are consistently of a lower intensity, such as strolling, gardening, golf, and low-impact aerobic activities [46]. Kim et al. (2022) underlined the importance and influence of light physical activity, which can help reduce mortality in older adults; thus, any level of PA is encouraged for mortality benefits [47]. Regardless of PA, regularly engaging in physical exercise can enhance the quality of life, promote good health, improve physical function, and mitigate the risk of falls in older individuals [42,48,49,50,51,52].

Nearly one-sixth of our population completely or greatly depended on their caregivers’ care. Meanwhile, the findings of previous studies in Austria (54.5%) and Poland (50.5%) presented higher percentages of older people who relied on caregivers to meet their needs [41,53]. This difference can be explained by the high number of people with mild and moderate dementia compared to severe conditions in our study, in which cognitive impairment remains mild, leading to lower dependence. When it comes to the levels of dementia, we also found that people with moderate and severe dementia had a higher level of care dependence than those with mild dementia. This result is consistent with a previous study conducted on nursing home residents in Germany, which revealed that care dependency was greater in those with dementia than in those without, and it grew stronger over time [54]. According to the results from a cross-sectional study carried out in Austrian hospitals and geriatric institutions, PLWD were significantly more care-dependent than those without dementia, according to all the items of the CDS [53]. Evidence from the literature demonstrated that dementia gradually impairs cognitive and behavioral functioning, which is the most important cause of care reliance [55,56,57,58]; therefore, patients with severe dementia require greater assistance from caregivers because of a lack of capacity to manage their physical and psychological needs. As explored in the study by Feenstra (2023), factors influencing PA in PLWD revealed potential barriers and facilitators; hence, addressing such problems is crucial in promoting physical activity and mitigating dependence [59]. Furthermore, engaging in exercise, as highlighted by Chen’s study, leads to better upper body strength, aerobic endurance, and balance in PLWD [60]. This underscores the positive impact of PA on functional abilities, potentially reducing dependence. People with moderate and severe dementia in our study were subject to higher levels of PA and consumed more energy than the other groups. This could demonstrate that people with more severe dementia are encouraged to participate in more exercise, representing the right focus of treatment attention at the treatment hospital.

We investigated the higher level of care dependence, which is one of the factors significantly related to less activity in PWD. In comparison, a study of hospitalized PLWD in the US showed that male gender and independence with ambulation were linked to higher activity levels [61]. Grand et al. (2022) indicated that PLWD experienced a range of symptoms related to cognitive, functional, and behavioral impairments; hence, one of the three basic clinical manifestations of PLWD was difficulties in ADL (domestic chores, personal hygiene, and food, dressing, eating, and bathing) [25]. Consequently, the patients progressively relied more on caregivers as they became more inactive in all their personal activities. To be specific, Astudillo-Garcia et al. (2016) investigated the contribution of dementia to disability, revealing a strong association between dementia and severe disability [62]. The individuals with dementia at baseline were more likely to have severe disability at follow-up, emphasizing the progressive nature of dependence in this population [62]. Dementia causes a decline in cognitive and behavioral abilities, making patients increasingly reliant on caregivers and, thus, less likely to engage in personal activities. Engaging in increased PA can have a detrimental impact, exacerbating health conditions and heightening reliance on caregivers.

Our findings also suggested that malnutrition is a significant factor that impacts the PA levels of PLWD. This was consistent with some previous studies in clinic settings in France and Italy, which demonstrated that individuals who were at risk of malnutrition exhibited worse cognitive function and more impairment in their ability to carry out ADLs compared to those who were adequately nourished [63,64]. PWD may experience a loss of their capacity to perceive their own hunger and thirst signals, an inability to feed themselves, a lack of interest or avoidance of food, difficulty in identifying familiar objects and perplexity, and dependence on others for aid with eating and swallowing (dysphagia), eventually leading to advanced dementia [65]. As dementia progresses, the need for assisted living increases due to the behavioral and psychological changes that occur, such as walking without purpose, worsening cognitive impairment, and difficulties in independently engaging in ADL [66]. Furthermore, malnutrition resulting in the loss of skeletal muscle mass increases stagnation and weakness, and reduces physical exercise [67].

Regarding the strengths of this study, this research contributes valuable insights specific to the Vietnamese context, addressing a critical gap in understanding PA levels and related factors in PLWD. However, this study’s limitations should be noted. First, the cross-sectional design may restrict our ability to establish causal relationships between PA and associated factors. Second, recall bias may occur due to the patients’ deteriorating memory and the caregivers’ inattention. Third, selection bias may exist due to the convenience sampling method used. To mitigate this, we recruited inpatient and outpatient participants to increase our ability to reach the target population. Finally, because of the limited sample size, we did not find significant differences in physical activity intensity between the dementia severity groups. Further studies should expand the scope of this study and increase the sample size to increase the representativeness of the population.

Based on the findings, several recommendations can be proposed. Enhancing the nutritional status of individuals with dementia can lead to an improvement in their physical activity. To mitigate malnutrition, it is imperative to prioritize the development of tailored dietary plans and nutritional care strategies for each individual. Moreover, caregivers should establish optimal circumstances for patients to engage in various activities independently. In addition, it is important to support PLWD in engaging in physical exercises appropriate for their physical status and intensity rather than remaining bedridden and reliant on the caregiver. In order to accomplish this, it is imperative to provide staff training, so we highly recommend that healthcare workers, as well as formal caregivers, participate in specialized courses that focus on the right care for individuals living with dementia.

## 5. Conclusions

A high percentage of the participants were classified as having low PA levels and were completely cared for independently. Higher levels of CD and being malnourished/at risk of malnutrition were factors that were positively related to the low PA level among the PLWD. Caregivers and healthcare workers should encourage PLWD to perform personal activities by themselves and personalize the development of tailored dietary plans and nutritional care strategies for individuals.

## Figures and Tables

**Table 1:** Socio-demographic characteristics and related factors of study participants (n = 63).

Characteristics	Frequency (n)	Percentage (%)	
**Gender**			
Male	20	31.8	
Female	43	68.2	
**Current occupation**			
Retirement	62	98.4	
Working	1	1.6	
**Currently living with**			
Family (wife/husband/child)	59	93.6	
Caregiver	1	1.6	
Live alone	2	3.2	
Other	1	1.6	
**Living areas**			
Rural	16	25.4	
Urban	47	74.6	
**Educational attainment**			
Below high school	26	41.3	
High school	12	19.0	
Above high school	19	30.2	
Unknown	6	9.5	
**The severity of dementia according to MMSE**			
Mild	33	52.4	
Moderate and severe	30	47.6	
**The nutritional status according to MNA**			
Normal	16	25.4	
Malnutrition and at risk of malnutrition	47	74.6	
	**Mean**	**SD**	**Min–Max**
Age (years)	74.7	7.3	51–94
Weight (kg)	55.5	8.9	33.1–73.2
BF (%)	32.4	7.3	17.1–45.7
BMI (kg/m^2^)	24.4	5.5	15.5–61.4
MMSE (score)	17.1	7.0	0–28

MMSE: Mini Mental State Exam; MNA: Mini Nutritional Assessment; BF (%): body fat percentage; BMI: body mass index.

**Table 2:** Physical activity levels of study subjects by severity of dementia (n = 63).

	Total (n = 63)	Normal and Mild Dementia (n = 33)	Moderate and Severe Dementia (n = 30)	*p*-Value
**Physical activity levels (n, %)**				
Low	22 (35.0)			
Moderate	41 (65.0)			
**Physical activity (min/week)**				
Walking (min/week)	856.3 ± 985.0	748.7 ± 595.9	994.5 ± 1335.1	0.25 ^M^
Moderate-intensity activity (min/week)	414.1 ± 296.0	322.5 ± 259.1	524.0 ± 327.8	0.28 ^T^
Vigorous-intensity activity (min/week) ^a^	135.0 ± 21.2			
**Energy expenditure (MET)**				
Walking (MET-minutes/week)	2825.6 ± 3250.4	2021.5 ± 2019.5	2297.4 ± 3965.8	0.13 ^M^
Moderate (MET-minutes/week)	1656.4 ± 1184.1	234.6 ± 650.5	349.3 ± 931.9	0.98 ^M^
Vigorous (MET-minutes/week)	1080.0 ± 169.7	29.1 ± 167.1	40.0 ± 219.1	0.93 ^M^
Total physical activity (MET-minutes/week)	2476.4 ± 3412.5	2285.1 ± 2202.1	2686.7 ± 4411.2	0.15 ^M^

**^a^** Vigorous-intensity activity (min/week) is shown for the total number of participants only. The figure for each group of dementia severity levels cannot be calculated due to the small number of participants. ^M^: Mann–Whitney test; ^T^: *t*-test. Data are expressed as mean ± SD.

**Table 3:** Factors related to low level of physical activity among study subjects (n = 63).

Factor		Low Level of Physical Activity		Crude OR (95% CI)	*p*-Value	Adjusted OR (95% CI)	*p*-Value
		No (n = 41)	Yes (n = 22)				
		Mean (SD)					
Age (years old)		73.7 ± 7.4	76.7 ±6.9	1.1 (0.98–1.15)	0.13	1.0 (0.95–1.16)	0.35
BMI (kg/m^2^)		24.4 ± 6.5	24.3 ± 3.0	1.0 (0.90–1.10)	0.91	0.9 (0.66–1.21)	0.46
Body fat percentage (%)		31.3 ± 7.1)	34.5 ± 7.5	1.1 (0.99–1.15)	0.11	1.2 (0.99–1.44)	0.06
CDS (score)		67.0 ± 14.4	50.6 ± 15.7	0.9 (0.90–0.97)	0.001 *	0.9 (0.88–0.99)	0.02 *
		**n (%)**					
**Gender**	Male	15 (36.6)	5 (22.7)	1	0.26	1	0.1
	Female	26 (63.4)	17 (77.3)	1.96 (0.60–6.40)		0.01 (0.01–1.48)	
**Dementia severity assessed by MMSE**	Normal and mild	26 (63.4)	7 (31.8)	1	0.02 *	1	0.21
	Moderate and severe	15 (36.6)	15 (68.2)	3.7 (1.24–11.15)		2.6 (0.58–12.07)	
**Nutritional status assessed by MNA**	Normal nutritional status	14 (34.2)	2 (9.1)	1	0.04 *	1	0.03 *
	Being or at risk of malnutrition	27 (65.9)	20 (90.9)	5.2 (1.06–25.44)		18.9 (1.40–256.11)	

* *p* ≤ 0.0–5: logistic regression; OR: odds ratio; CI: confidence interval; MMSE: Mini Mental State Exam; MNA: Mini Nutritional Assessment; CDS: Care Dependency Scale; BMI: body mass index. Quantitative variables are expressed as mean ± SD, qualitative variables are expressed as n (%).

## Data Availability

The data presented in this study are available on request from the corresponding author (due to privacy reasons).

## Supplementary Materials

The following supporting information can be downloaded at https://www.mdpi.com/article/10.3390/geriatrics9030057/s1, Table S1. Median and interquartile range (IQR) of BMI and some physical-related variables of study subjects (n = 63); Table S2. Mean score of each component of Care Dependency Scale (CDS) (n = 63).

## Abbreviations

95% CI: 95% confidence interval; ADL, activities of daily living; BF%, body fat percentage; BMI, body mass index; CD, care dependency; CDS, Care Dependency Scale; DSM-V, Diagnostic and Statistical Manual of Mental Disorders, Fifth Edition; IPAQ, International Physical Activity Questionnaire—Short form; IPAQ, International Physical Activity Questionnaire; MET, energy expenditure; MMSE, Mini Mental State Exam; MNA, Mini Nutritional Assessment; MNA-LF, Mini Nutritional Assessment—Long Form; NGH, National Geriatrics Hospital; OR, odds ratio; PA, physical activity; PLWD, people living with dementia; SD, standard deviation.
