# Supplementary material for: An Assessment of Physical Activity and Risk Factors in People Living with Dementia: Findings from a Cross-Sectional Study in a Long-Term Care Facility in Vietnam
Source: Geriatrics (Basel). 2024 Apr 29;9(3):57. doi: 10.3390/geriatrics9030057 (PMC11130955; doi:10.3390/geriatrics9030057)
Supplement: Supplementary file 1 [file geriatrics-09-00057-s001.zip › Table S1.pdf]

Table S1. Median and interquartile range (IQR) of BMI and some physical-related variables of study subjects (n=63).

Variables	**Median**	**Q1**	**Q3**	**IQR**
BMI	23.7	21.9	25.7	3.9
Minutes per week of walking	420	210	1260	1050
Walking (MET-minutes/week)	1039.5	0	2772	2772
Moderate (MET-minutes/week)	0	0	0	0
Vigorous (MET-minutes/week)	0	0	0	0
Total physical activity (MET-minutes/week)	1386	66	3066	3000
