# Supplementary material for: An Assessment of Physical Activity and Risk Factors in People Living with Dementia: Findings from a Cross-Sectional Study in a Long-Term Care Facility in Vietnam
Source: Geriatrics (Basel). 2024 Apr 29;9(3):57. doi: 10.3390/geriatrics9030057 (PMC11130955; doi:10.3390/geriatrics9030057)
Supplement: Supplementary file 1 [file geriatrics-09-00057-s001.zip › Table S2.pdf]

Table S2. Mean score of each component of Care Dependency Scale (CDS) (n=63).

Score	Mean	SD
Eating and drinking	4.22	1.29
Continence	4.38	1.1
Body posture	4.1	1.2
Mobility	3.9	1.4
Day/night pattern	4.24	1.32
Getting dressed and undressed	4.03	1.38
Body temperature	4.33	1.22
Hygiene	3.94	1.47
Avoidance of danger	4.24	1.25
Communications	4.16	1.15
Contact with others	4.1	1.23
Sense of rules and values	4.17	1.19
Daily activities	4.19	1.32
Recreational activities	3.7	1.53
Learning activities	3.6	1.47
